# Perceptions of Communities of Practice and Sense of Belonging: Focus Groups of Academic Pediatric Faculty

**DOI:** 10.7759/cureus.63605

**Published:** 2024-07-01

**Authors:** Margaret P Huntwork, Myo T Myint, Emma Simon, Bonnie Desselle, Amy M Creel

**Affiliations:** 1 Clinical Immunology, Allergy, and Rheumatology, Tulane University School of Medicine, New Orleans, USA; 2 Child and Adolescent Psychiatry, Tulane University School of Medicine, New Orleans, USA; 3 Office of Medical Education, Children's Hospital New Orleans, New Orleans, USA; 4 Pediatric Critical Care Medicine, Louisiana State University Health Sciences Center School of Medicine, New Orleans, USA

**Keywords:** focus groups, faculty engagement, faculty development, sense of belonging, communities of practice

## Abstract

Background: Providing the opportunity for collaboration around a central purpose to improve skills and exchange knowledge, the Community of Practice model can be useful for faculty development. A sense of belonging enhances the engagement in communities. Yet, the barriers and contributors to academic medicine faculty’s sense of belonging in communities are not as well explored.

Methods: Through focus groups with 21 academic pediatric faculty conducted between January and March 2023, this qualitative study examined knowledge of Communities of Practice and the factors that affect sense of belonging and engagement. The authors iteratively coded transcripts to generate themes.

Results: Community accessibility; opportunities for active engagement; working under a clear, shared purpose; and personal interactions enhanced faculty sense of belonging. Barriers to engagement included competing demands, process challenges, and uncertainty.

Discussion: Study results suggest strategies for the promotion of faculty sense of belonging and engagement in Communities of Practice. Consideration of contributors to a sense of belonging may enhance efforts to design and improve engaging faculty development programs.

## Introduction

Academic literature increasingly describes a Community of Practice (CoP) as a beneficial model for the faculty development of medical professionals [1-5]. Evidence suggests that successful implementation of the CoP model involves faculty engagement, which is enhanced by a sense of belonging [6-8]. Yet, faculty's perspectives on this remain underexplored. As a community striving to better reflect and represent a diverse patient population, academic medicine must consider how to support a sense of belonging for all faculty.

A CoP can be defined as “a persistent, sustaining social network of individuals who share and develop an overlapping knowledge base, set of beliefs, values, history and experiences focused on a common practice and/or mutual enterprise” [9]. The community nurtures faculty members as their professional identities form and evolve [2]. Full participation in a CoP has been described as “a mode of belonging” [2,3,10]. Further, the fundamental psychological need of a sense of belonging [11,12] increases confidence, professional satisfaction, and professionalism [13-15], and is a key component of a thriving community [6].

Best practices in academic medicine rely on development of faculty, which can occur through formal (e.g., courses and lectures) and informal (e.g., daily interactions with colleagues) approaches [1]. Published studies provide insight on the CoP model for faculty development, but guidance for actively supporting faculty sense of belonging within a community is lacking [1,4]. Our hospital-based faculty development program strives to engage faculty with different backgrounds, academic affiliations, and specialties in an inclusive community. Thus, in this phenomenological study, we aim to better understand the contributors and barriers to academic medical faculty's sense of belonging in a CoP by exploring their perceptions and experiences.

## Materials and methods

Study design

We developed questions exploring faculty perceptions and experiences with medical Communities of Practice. Social cognitive theory informed the project, and we chose a phenomenological approach to gain insight into the lived experiences of faculty [16]. We selected focus groups to encourage sharing of experiences and prompt recollections among participants and did not request post-focus-group reflections or surveys to limit the participation time required of faculty for this study. We obtained Internal Review Board exemption status and ethical clearance from all three participating institutions: Louisiana State University (LSU), IRB# 4730; Children’s Hospital New Orleans, IRB# 22-085; Tulane University Human Research Protection Office, IRB# 2022-1814.

Participants and recruitment

We recruited faculty participants from the three participating institutions (two schools of medicine and one hospital employment group) that practice in one academic, free-standing, quaternary care children’s hospital. Emails and departmental newsletters announced a call for participation in a focus group regarding communities of practice. We used purposeful criterion sampling to include any medical faculty for whom our hospital is their primary patient care location. 

A total of 21 faculty participated in five focus group sessions, with three to six participants per session. We did not exclude any participants as all met the inclusion criteria; faculty practicing with our children's hospital as their primary clinical site. Focus group questions are included in Table 1, and Table 2 shows the results of brief written demographic questions that participants voluntarily completed prior to each session.

**Table 1:** Questions posed to each of five focus groups of academic pediatric faculty.

Focus Group Questions
What professional groups do you identify with?
How do you engage with these groups? How would you describe your level of engagement?
Do you experience a sense of belonging with the group(s)? Why and why not?
How did you start engaging with the group(s)?
The definition of a community of practice is a group of people who share a concern or a passion for something they do and learn how to do it better as they interact regularly. Would you describe any of the groups you engage with as meeting this definition?
Would you describe any groups as your community of practice?
Do you obtain your professional development from your community of practice?
Do you feel there is sufficient opportunity to obtain your professional development from our hospital? What are the barriers?

**Table 2:** Demographics of focus group participants from a 2023 qualitative study of academic medical faculty experiences of sense of belonging and understanding of communities of practice.

Primary Appointment (n=21)		Career Level (n=21)		Specialty (n=21)		Gender (n=21)	
Institution 1	1 (5%)	Early (0-8 years)	12 (57%)	General Pediatrics	1 (4%)	Female	17 (81%)
Institution 2	15 (71%)	Mid (9-19 years)	5 (24%)	Pediatric Subspecialty	20 (96%)	Male	4 (19%)
Institution 3	5 (24%)	Late (20+ years)	4 (19%)				

Data collection

We utilized pseudonyms to protect identities, and the facilitator provided instructions to promote comfort in sharing personal experiences and feelings.

One author (E.S.) conducted all five semi-structured focus groups from January 2023 to March 2023, each lasting approximately 60 minutes. A hybrid format via Zoom (San Jose, CA, USA) was utilized for faculty unable to attend in person (18 in person and three virtual). Sessions were audio-recorded and transcribed verbatim using a professional transcription service (Scribie, San Francisco, CA, USA).

Data analysis

We immersed ourselves in the transcripts to better understand participants’ lived experiences using a phenomenological framework outlined by Braun and Clarke [17]. All five authors participated in the process of transcript analysis using an iterative group process of review, discussion, and reconciliation for generation of themes. At least three investigators were present for each analysis session. Investigators utilized Dedoose (Manhattan Beach, CA, USA) during analysis of transcripts and reflections.

Author positionality and reflexivity

We are members of a hospital-based research community focused on education and scholarship across the continuum of medical training and practice. The team includes four pediatricians (three subspecialists and one triple board) and one medical education administrator. All hold positions related to medical education and primary appointments at one or more of the three represented institutions.

We acknowledge that our positions and perspectives can influence this work. We integrated frequent reflection, discussion, and consideration of our own individual and group experiences into all phases of this study. To provide consistency and to avoid the potential power dynamic that could be associated with faculty leading the focus group sessions, a single investigator (E.S.) who is an administrator and does not supervise or evaluate any faculty, conducted all sessions. We analyzed the data as a group to minimize individual biases [18].

## Results

Knowledge and perceptions of communities of practice

Participants expressed limited pre-existing familiarity with the term “Community of Practice,” but identified various groups they participate in as meeting the provided definition.

“I think I've heard of [Community of Practice], but not in a deep, meaningful way.” [Focus Group (FG) B, speaker (sp) 1]

“Yeah, agreed. I had never heard of [Communityit of Practice] before, but immediately thought of my group practicing medicine, whenever I saw it.” [FG A, sp 1]

“No, I had not heard of communities of practice.” [FG B, sp 4]

Faculty vocalized the importance of a CoP, specifically due to their educational value.

“Especially in current medicine, it keeps us updated; it keeps us practicing safe medicine. The community of practice is integral to medicine today and it's only gonna become more important as time goes on.” [FG B, sp 2]

“Hopefully I'm a[n] okay doctor, but I wouldn't be the doctor that I am without my community of practice.” [FG D, sp 3]

“Because we can't be experts in everything and with the knowledge growing so much, these independent but then overlapping communities of practice make us not better just for patients, but for the entire community of medical training and research and growth.” [FG B, sp 1]

Professional growth and academic and career opportunities were also valued benefits of participation in a CoP.

“If it weren't for the group and belonging to the group, I wouldn't have a current dream job… I wouldn't have ever known that this opportunity even existed.” [FG B, sp 2]

“An invitation to be part of that conference. And then out of that…, an invitation to teach at the conference.” [FG C, sp 5]

Respondents lacked formal knowledge of the term CoP or the educational theory behind it. However, they “know it when they see it,” and importantly, they valued it.

Contributors

Faculty experiences of sense of belonging are enhanced by features of the CoP, including 1) opportunities for active engagement, 2) enhanced accessibility, 3) clear shared purpose, and 5) personal interactions.

Engagement in the CoP strengthened faculty’s sense of belonging. Participants reported that active roles, not merely passive attendance, contributed to positive experiences.

“How much you give is how much you're gonna get back.” [FG D, sp 4]

“Sense of belonging, communities of practice, you get out of it what you put into it.” [FG B, sp 1]

Accessibility facilitated participation and engagement. Factors such as the format, location, and timing of meetings, as well as the types and frequency of communication, influenced accessibility and therefore affected faculty sense of belonging. 

“The fact that Grand Rounds are more accessible now...Being able to not only be in person at it… we travel to multiple different clinical sites, and so being able to access it at any point from anywhere [virtually]…has been really helpful.” [FG A, sp 3]

“Proximity. It's all right here.” [FG E, sp 1]

“Whether it's our group text chats… informal chats, weekly meetings, or even the opportunities to do these professional development courses…” [FG A, sp 3]

Alignment with the purpose of a CoP contributed to faculty engagement and sense of belonging. 

“We get to focus on the thing that kinda made us all start in the first place. So, it's kind of nice to have a little forum where we just get to be excited about our sub­specialty and nothing else really has a place for that one hour.” [FG A, sp 1]

“There's no question [that] there's a shared purpose of passion in that particular group. And it's reinvigorating… And so, it's nice to go and get a little bit of a boost.” [FG E, sp 1]

“[A] wide variety of people from our department, many that I've admired and looked up to for years, and people my age, and people more junior…all there with this one purpose…” [FG A, sp 1]

Personal interactions and relationships were described as integral to both initial and sustained faculty engagement. Faculty noted an environment welcoming of their ideas and contributions, inclusion of time for both formal and informal interactions, longitudinal relationships, and specific invitations to join as contributing to higher levels of engagement. Additional description of these and other types of interactions with illustrative quotes are in Table 3.

**Table 3:** Personal interactions and relationships contribute to faculty sense of belonging and engagement in Communities of Practice. Focus group participants in a qualitative study of faculty (N=21) at a free-standing children's hospital describe the ways in which interactions are perceived as positive contributors. FG: Focus Group, sp: speaker

Contributor to Sense of Belonging	Illustrative Quotes
Welcoming ideas and contributions: Faculty value being able to express ideas and offer suggestions.	“I also feel like people value my input and ideas” [FG A, sp 2]
	"Since I've joined every year, there's been a new hire... anybody new that comes in brings with them this whole fund of knowledge and their style of doing things, which we learn from...I really appreciate that. Like the two people that came after me, I still learn things from them." [FG C, sp 1]
Growing together: Time spent together as groups launch or encounter change enhance individuals’ sense of belonging	“The group that I'm involved in, it started as an email about interest in developing a program, and then that turned into attending meetings. And then they just started having so many people at the same time as me and ... we all kind of grew together.” [FG A, sp 1]
	“There's just a lot of collaboration, communication between the group [members] but I think we all learn a lot from those” [FG D, sp 2]
Mentors and recommendations: If a mentor recommends that an individual joins a group, there is increased sense of belonging	“For me, it was…a recommendation…[I] was recommended by one of my mentors to join.” [FG E, sp 3]
	“I was encouraged by my mentor to go to the national meeting and get involved…And now it's a meeting I won't miss.” [FG E sp 1]
Leadership: When individuals look up to the leadership, sense of belonging is enhanced	“I also enjoy when there's professional development…led by people that I've always admired as physicians and role models.” [FG A, sp 3]
	“Leadership in the groups…makes a big difference in how I view those groups and how I view them being successful or view them as something that I want to continue to participate in. [FG A, sp 2]

“I have different opinions and thankfully those opinions have been met with …with open-mindedness, which is very nice.” [FG C, sp 5]

“Other like-minded people and people who have similar interests…[They] find a conference or an opportunity and then tell [another] about it because…that person has mentioned before that they have an interest in some specific project or idea...That's helpful in that people do know you and they may know that you have an interest in X, Y, Z and so, the opportunities to find out about some of those educational opportunities are better because of the people around you.” [FG A, sp 2]

“I do feel a sense of belonging…has to do with the length of time I've been here, and [I've] developed some different relationships.” [FG D, sp 4]

Opportunities for contribution, ease of accessibility, shared purpose, and personal interactions enhanced faculty sense of belonging and engagement. Focus group responses highlighted the frequent intersection of more than one positive contributor in communities where they experience a high level of engagement.

Barriers

Faculty reported competing demands, process challenges, and uncertainty as barriers. Whether alone or in combination, these barriers decreased attendance, engagement, and sense of belonging.

“Compared to 10 years ago when I first entered medicine…it's getting exponentially more prominent every year. It is a higher and higher push from corporatized medicine... We're prioritizing patients but there's some lacking in structures for physician support and physician well-being. And professional development because there's just no time. And I do have to go to another meeting.” [FG D, sp 4]

Competing demands for faculty attention included clinical responsibilities, time for family, and personal care. Additionally, faculty reported feelings of maximized cognitive load as a barrier to engagement.

“Being on service and having a professional development opportunity offered only once...And often...in the middle of the rounds...or at noon when theoretically we should be done but on...service or call days with patient care, it's so unpredictable... scheduling conflicts with patient-safe care probably is the biggest barrier for me.” [FG D, sp 3]

“I prioritize my kids over evening meetings.” [FG B, sp 4]

“The planning, the organization, mental preparedness of all of these things, that's an unmeasured cost potentially…adds to the day. All of the other expectations…, I just need to breathe and don't have the mental time to... get one more thing on…[my] plate.” [FG D, sp 1]

“Our clinical and educational requirements and expectations have continued to grow. The time commitment is definitely a really big deal. There are some things that I would like to do and… learn… There's not enough time and the scheduling to do it.” [FG A, sp 2]

“As physicians, our community is committed to prioritizing patients above all else... potentially here in academics, it's patients above all else followed by our trainees... and then all the other tasks you have and then finally your own self... That is not necessarily the correct order, but I think that as physicians, that's often the order that we place things in in terms of prioritization and... that's why it's hard to find time.” [FG D, sp 4]

Technology challenges, lack of funding, difficulty with reservation systems, locations, and time of meetings were some of the process challenges.

“Technology. Huge challenges trying to understand the computer system and the networks associated.” [FG E, sp 4]

“And sometimes that little extra step is more than I have time for or have the mental capacity to figure out what my password is.” [FG D, sp 4]

“Another barrier is funding… the paperwork and the red tape and how to figure out how to get through some of those things…” [FG A, sp 2] 

“That geography's important. And if there's Zoom options, but you're the only one on Zoom, then it's almost like not really being there.” [FG E, sp 1]

Faculty perceived uncertainty about a CoP or the opportunities it offered as barriers to engagement. Specific barriers reported include lack of information about the existence of a community and the purpose and benefits of belonging.

“I don't know if [my institution] specifically offers them…Or maybe, I just don't know.” [FG C, sp 1]

“[Lack of information] on how things work, and how things maneuver or how you communicate with each other and so on… Very unstructured…, so it's been a challenge to try and connect for me.” [FG E, sp 4]

“[Am I] committed to a mission that not everyone is committed to?” [FG D, sp 4]

Suggested strategies

Participant responses suggested strategies to those planning and leading faculty development communities. Embracing described contributors and avoiding barriers may support faculty sense of belonging and enhanced engagement. See Table 4 for strategies to consider paired with illustrative quotes.

**Table 4:** Strategies to promote faculty sense of belonging in a Community of Practice based on a 2023 qualitative study of academic pediatric faculty from three institutions (n = 21). FG: Focus Group, sp: speaker

Strategy	Suggested Action	Illustrative Quote
Educate faculty about communities of practice	Explicitly state that the group is a Community of Practice and define what that means. Reflect on the value of belonging to a Community of Practice	“Communities of practice was not a term that I had really heard or been familiar with prior to today… but I do think it makes all the difference in the world and then having worked at different institutions and been now within different communities of practice,...it has made or broken my experience and sense of belonging within our department… or sometimes within the institution.” [FG D, sp 1]
Invite new ideas and contributions	Cultivate a community environment that welcomes new members, ideas, and contributions	“Meaningfulness in being able to contribute or be part…[of] a group that respects your opinions.” [FG D, sp 3]
Support interactions and relationship building	Personalize invitations to join the group; cite strengths that individuals will bring, begin meetings with introductions of group members	“It was recommended by one of my mentors to join…Those opportunities that I…have been most connected with, have been [from recommendations]...” [FG E, sp 3]
	Leaders are visible, approachable, and act as mentors and sponsors	“The leadership part is really huge…people I view as mentors or as successful leaders, when they are involved in a group, it makes me want to gravitate towards that group more.” [FG A, sp 2]
	Leave time in meetings for informal networking	“[I] enjoy in person get togethers and really value that.” [FG C, sp 2]
Optimize accessibility	Select meeting times and plan meeting frequencies that do not interfere with other responsibilities (personal or professional)	“It's hard to feel a sense of belonging…when group things are held at times that may not be conducive to someone with a family,…dinner time, bedtime. There's sometimes meetings that happen at 6:00 PM and last till 8:30 PM…Time and attention to those details is important when planning meetings and planning sessions and can be a hindrance to some who can attend. The more it happens that way…every single time the meeting occurs at that time, the more the individual may not be able to attend, the more dissatisfied that individual grows with the process.” [FG D, sp 4]
	Protect time for meetings	“[The biggest barrier to participation is]…lack of time, lack of protected time.” [FG D, sp 3]
	Offer hybrid options in addition to convenient meeting locations. Engage faculty, including those participating virtually	“Zoom options have been really helpful.… A hybrid option really helps for when you're not onsite… which I spend at least half my time somewhere else.” [FG C, sp 5]

## Discussion

Cruess et al. recommend making “communities of practice an explicit part of the curriculum” [2]. Yet, our study suggests academic pediatric faculty lack familiarity with the term Community of Practice despite increasing use of this term in the medical literature. Notably, once provided with a definition, study participants characterized some groups and organizations they engage with as Communities of Practice. The roles these communities play in enhancing faculty development and up-to-date patient care was an additional theme. As such, does promoting knowledge and use of the term matter if faculty “know it when they see it”? Boudreau et al. suggest that it does; defining the term CoP for faculty may promote self-reflection and validation of one’s commitment to and passion for medicine [19]. 

As Steinert so eloquently wrote, “we need to help our colleagues value the community of which they are a part (e.g., by celebrating its existence, members, and resources) and find community (e.g., by building new networks, creating opportunities for exchange and support, and sustaining relationships)" [1]. Our results further the conversation on the importance of the socialization component and the experience of a sense of belonging for faculty in a CoP. Participants reported that both initial engagement and longitudinal participation were affected by member interactions as well as experiences with those in community leadership. Personal interactions, many of which might be considered informal, were reported to increase engagement with formal communities, and vice versa. This suggests that plans for faculty development may benefit from intentional integration of opportunities for personal interactions. Additionally, leaders should be visible, approachable, and should act as sponsors [8]. Like Chandran et al. [20], our results suggest personalization of invitations and opportunities enhance engagement. Building in explicit time for participant interaction may support networking, mentoring and collaboration.

Study participants emphasized the importance of the ability to contribute. Opportunities to facilitate the education or functioning of the community, as well as to participate in outward-facing projects contribute to a sense of belonging. Further, experiences of contributing to a shared purpose, and being able to further that purpose through meaningful projects offer the ability to engage in full participation. Communities of Practice center around a “common practice and/or mutual enterprise” [6]. Our results reinforce that a clear, explicit statement of the community's purpose may avoid uncertainty and the decreased engagement that can accompany it.

In this era of advancing technology, it is notable that the theme of accessibility is a contributor to engagement, but some aspects of using technology act as a barrier to participation. Virtual and hybrid options for faculty development have been shown to increase attendance [21]. Our results affirm facilitation of participation of geographically distanced faculty. Importantly though, participants emphasized that virtual participation does not always equate to engagement. Some participants expressed feelings of alienation from the group related to remote access. In planning events and communications, consideration of optimizing accessibility for increased engagement rather than just increased attendance should be considered. Conversely, technology may magnify process challenges. Faculty expressed frustration with registration systems and passwords and reported avoiding some opportunities due to technological barriers. 

As physicians face increasing demands [22] and the integration of multiple professional identities [23], Communities of Practice may struggle or flounder without minimization of barriers. If a community is seen as competing with other aspects of work, particularly patient care, then it may not be prioritized. Timing must also be carefully considered as imposition on clinical duties, family or personal wellness time may decrease engagement.

Existing works document that enhancing a sense of belonging bolster the goal of faculty progression from peripheral to full participation [1,2,24]. Our results affirm the importance of sense of belonging and add to the existing literature by characterizing contributors and barriers. Further, participant responses suggest potential strategies for the active promotion of faculty sense of belonging in a CoP.

Strengths and limitations 

A strength of our study is the inclusion of faculty from a variety of inpatient and outpatient pediatric specialties, with differing academic affiliations, and levels of experience ranging from early to late career. Thus, our participants offer a wide variety of perspectives, but demographic representation is notably skewed toward early career and female physicians. Faculty with differing demographics and backgrounds may regard sense of belonging in communities differently, making this an important avenue for future inquiry. The risk of bias that is associated with self-selection to participate in a research study is another limitation that may affect our results. 

Future directions

Future studies are needed to add depth and nuance to our understanding of the health and success of faculty development communities. Moving beyond attendance to evaluate the reciprocity between the individual and the community may be a good starting point. Consideration of outcome and process measures that include faculty sense of belonging may be beneficial.

## Conclusions

As faculty development efforts embrace the CoP model, consideration of member sense of belonging may enhance engagement. Our study reflects a need for additional faculty education on this model and offers insight into facets that contribute to faculty engagement. Participants report a sense of belonging as strengthening engagement, and detail ways in which engagement supports a sense of belonging. Faculty’s sense of belonging can be enhanced through cultivation of accessibility, opportunities to contribute, shared purpose, and interpersonal interactions. Further, the description of barriers provides guidance for limiting obstacles. Consideration of these elements and their seeming reciprocity may aid in the creation or enhancement of faculty programming that is ever closer to achieving inclusivity and full participation.
